# Round the Hospitals

**Published:** 1920-04-10

**Authors:** 


### ROUND THE HOSPITALS.

The King has awarded the Royal Red Cross to the following ladies of the nursing services in recognition of their valuable services in the Field (*a*) with the British Army of the Black Sea (dated June 3, 1919):—Bar to the Royal Red Cross: Miss I. M. Turner, R.R.C.; A. Matron, Q.A.I.M.N.S. (R.). Royal Red Cross, 2nd Class: Staff Nurse C. I. Cassidy, Sister F. G. Hobbs, Q.A.I.M.N.S. (R.); Sister E. V. Boult; Staff Nurse L. E. Leigh; Sister L. Lister; Sister C. M. Paterson, T.F.N.S. (*b*) With the British Forces in Egypt (dated June 3, 1919):—Royal Red Cross, 1st Class: Sister A. I. Sayer, Q.A.I.M.N.S. (R.). 2nd Class: Sister I. M. Reynolds and Sister A. Taylor, Q.A.I.M.N.S.

**ROUND THE HOSPITALS**—(*continued*).

(R.). (*c*) With the British Forces in Mesopotamia (dated June 3, 1919):—Bar to the Royal Red Cross: Miss A. L. Earle, R.R.C., Matron T.F.N.S.; Royal Red Cross, 1st Class: Sister E. C. E. Lindsey, A.R.R.C., Q.A.I.M.N.S. 2nd Class: Sister B. Crowley and Sister H. McLeonard, Q.A.I.M.N.S. (R.); Misses M. Hearn and A. B. Ross, attached Q.A.I.M.N.S. for India; Sister B. Brown and Sister E. A. Mincaster, T.F.N.S. The King has been graciously pleased to award the Royal Red Cross, 1st Class, to Miss Minnie Bryne, T.F.N.S., for gallantry and devotion to duty at the 2nd Northern General Hospital, Leeds, on January 1, 1920, when, through an unforeseen circumstance, a patient's bed was set on fire. With great presence of mind Staff Nurse Byrne endeavoured to smother the flames, and while doing so her own clothing caught fire. She succeeded nevertheless in removing her patient to another bed though suffering extensive burns herself.

The King and Queen paid a cheering visit last week to the Hospital Home for disabled soldiers at Gifford House, Roehampton. It is one of the institutions where sufferers from spinal complaints are treated, and, as all nurses know, the greatest importance attaches to keeping the mental atmosphere free from depression or worry and a good current of hope stirring. The visit of Their Majesties was an event calculated to raise the spirits of patients and staff alike, and their thorough inspection of the institution gave pleasure to all, for every detail counts in their expert examination. The Queen's individual interest in the pursuits of the wounded gave rise to a charming incident, for noticing with her quick eye that one of the patients in a bath-chair was in possession of a camera she posed for him and begged for a copy of her picture to be sent on to her.

It is good news that an improved scale of salaries for the Nursing Services of both the War Office and the Ministry of Pensions is in course of preparation and will shortly come into force. The alterations are long overdue. The system of bonuses, with its implied stipulation that when prices fall the salaries will be reduced, is eminently unsatisfactory and unsettling; and apart from this the general rates of pay in the Services, which from their honourable associations rank as leading the way in the profession, have long been such as to discourage nurses since peace was declared from belonging to them. The importance of setting a high standard by the Government employers of nurses has not hitherto been fully grasped, but we trust the event will show it is now fully realised.

Fine work was accomplished by the American Red Cross Commission to Siberia, withdrawn last February. Their sphere of operations covered a stretch of 5,000 miles, from Vladivostock to the Ural Mountains. There was a chain of eight hospitals, with 152 nursing sisters, 42 aids, and a large complement of doctors and relief workers. They equipped and maintained an anti-typhus train, which travelled back and forth along the Trans-Siberian Railway. They established fourteen hospitals for sick and wounded Czech and Russian soldiers. They built barracks for the refugees and provided them with occupation. Many of the younger women and girls were employed in Red Cross hospitals, and a four months' course of first aid and nursing was started for them, which proved of the utmost educative value to the Russian girls. One of the splendid achievements of the Commission was the rescue of several hundred homeless children found in the Ural Mountains in a state bordering on savagery, lost from their parents. Their ages ranged from three to sixteen, and they were gathered into a colony, till they could be transferred to orphanages on Russian Island. Miss Clara Noyes, R.N., gives an excellent account of the activities of the Commission in the *American Journal of Nursing* for March.

The latest news of the *Daily Telegraph* Shilling Fund during the holidays shows it to be full of vitality and going strong. The Eccentric Club have sent in a further sum, which raises their total contribution to £315. The response to the appeal made by the *Leicester Mail* continues to elicit a good response, and shows contributions from the Duke of Rutland, Viscount Churchill, and well-known business firms in Leicester. The collections made by officers and non-commissioned officers in both the Navy and the Army are tokens of the high regard in which the nursing sisters are held in the Services, and we trust these may long continue to drop in from various parts of the world. "Ex-nurse" Crosbie, who has done such excellent work for the Fund, ought to number hundreds of disciples walking in her steps.

It has transpired that great disorganisation and confusion prevailed in every department of the Red, or Communist army in the Rhine during the recent rising. In particular the "Red" nurses were attacked by panic and fairly fled from the scene of action. Stoppage of pay and food shortage damped down the enthusiasm with which the movement started. The head of the medical service was to be seen crying out in wrath at the forlorn group of Red Army nurses, deserters from their post at the front, "and a beautiful black-haired girl with flashing eyes was emphasising the Doctor-General's reproaches in fiery words to the tearful nurses. Finally the doctor tore up their blue cards and threw the scattered fragments into the air."

Colchester appears to be very unfortunate in the choice of its Guardians. A scandalous state of affairs was exposed in their management of the Poor-law Infirmary by Dr. Philip Laver, medical officer, who attended a recent meeting of the Guardians in order to tell them the truth about the Infirmary. The place is terribly under-staffed and the over-worked probationers are shockingly badly paid, for the Guardians have steadfastly refused to

**ROUND THE HOSPITALS—(*continued*).**

give any war bonus. There are some 120 people in the Infirmary, of whom eighty-three are bed-ridden. To attend on these cases the Guardians have provided one certificated nurse and "about three wardmaids." The Ministry of Health has indicated that they need seven nurses. Fortunately matters have come to a crisis, because the helpers, who are indiscriminately alluded to as "the girls," "the nurses" and "the wardmaids," are being compelled to work eighty-one hours a week and have threatened to leave. A motion that the Board should go thoroughly into the matter at a special meeting was carried, and the results will be awaited wth some interest.

It would be a calamity to the County if the Training Home of the West Riding County Nursing Association were to come to an end, but it is in a very parlous state. It is doing excellent work in training and supplying thoroughly competent nurses and midwives throughout the county, and especially for the rural areas. The training centre was worked at a loss last year of £330, and for a time it seemed inevitable that the work should be abandoned. However, a more cheerful mood pervaded the supporters at the annual meeting on March 26 at the Leeds Church Institute. The West Riding County Council helps with the training fees to the extent of nearly £700, and the seventy-three affiliated associations are to be asked to increase their affiliation fee from one to three guineas. A strong financial committee was appointed to devise means for making both ends meet, and we trust that, with more publicity, the Nursing Association will meet with more support in the West Riding.

Some unusually interesting speeches were made at the annual meeting of the Birmingham and Midland Ear and Throat Hospital, when the Bishop of Birmingham presided. In spite of the fact that the ordinary income has increased by £435, the excess of expenditure was over £1,000 last year. The Bishop, speaking of State aid and rate aid as a terror, not only to the life of the hospital but to the taxpayer, said the real remedy was that everyone should bear his part and all givers should help to teach others to give. On the nursing side they are trying the experiment of getting V.A.D. nurses to come in during the day, when the rush of work is on, to help in relieving the regular staff. Mr. Ekin said if it were only known more widely how much the hospitals were understaffed as regards nurses he believed they would get more ladies to come forward, particularly if they could arrange for them to take part-time duty.

Miss E. M. Bann, who has been matron of the M.A.B. Brook Hospital for twenty-three years, has resigned her office, in obedience, we notice with regret, to doctor's orders. She contracted out of the Poor-law Officers' Superannuation Act, passed the year she became matron, but the Board has power to award an annual allowance under the Act of 1864. The amount in respect of twenty-completed years of service is £86 12s. 3d. per annum, which sum has been granted subject to the sanction of the Ministry of Health. Miss Bann is a notable figure among fever-hospital matrons, and will be grievously missed by all who have had the privilege of working with her and under her.

Tottenham people appear to be imperfectly informed of the benefits available for them in their District Nursing Association. The Chairman, Mr. F. W. Drewitt, expressed surprise at the annual meeting that the people should be content to remain in ignorance as to the existence of the Association. This was really rather naive, for a grain of imagination would have shown how impossible it is for new residents in any locality to come unaided to the knowledge of a society whose very existence is hidden from them. More publicity is needed, which would speedily strengthen the finances of the Association. We suggest that the Committee have a leaflet left at every house giving information about the nurses and asking for support, with a notice that a further call will be made within a few days to receive contributions or give further information.

In 1918 the Essex County Council made an agreement with the Essex County Nursing Association regarding the provision of nurse-midwives in the county. Recently County Council representatives have attended a conference with the Association, when the question of salaries of the district nurses and district nurse-midwives was discussed, and it was decided to put the following arrangements into force:—*Junior Nurses*: Salaries to be increased to £100 per annum, which would be the minimum salary for this class of nurse. *Senior Nurses*: The salaries of these nurses to be also increased to a rate somewhat above those of the junior nurses. *Superannuation Scheme*: A superannuation scheme should be commenced, and the proposals outlined should be submitted for actuarial and legal approval. The present scheme provides for a contribution in respect of each nurse of 2s. 6d. per week, one-half to be paid by the nurse and one-half by the Association. *Organiser*: As the inspection of midwives will be taken over by the County Council as and from April 1, 1920, it will be necessary in the interests of the Association to retain the services of a lady as organiser, whose salary, with travelling expenses, will, it is estimated, amount to £200 per annum.

An important decision was given last week by Mr. A. Steele Sheldon, the Registration Officer, at a sitting of the Wimbledon Borough Registration Court. It concerned an application from thirty-nine nurses, connected with five local hospitals, for the Parliamentary and local government votes. They claimed the votes as occupiers of bedrooms in their respective institutions by virtue of their employment. Mr. Sheldon, however, rejected their claim. He declared that in view of the discipline which must prevail in well-ordered hospitals, it

**ROUND THE HOSPITALS**—(*continued*).

could not be contended that a nurse was permitted to exercise in regard to her bedroom the ordinary rights which a man (or woman) exercised in respect of his own dwelling-house. This is the crux of the whole situation and there is undoubtedly considerable difficulty in proving analogy between the nurses' room in hospital and her room, say, in a private flat. There are a dozen things which a woman would feel at liberty to do in her own apartments which no matron would or ought to tolerate in a resident of the Nurses' Home, keeping the door locked with a private key, for instance, inviting friends in to a meal, additional articles of furniture, and so on. So large a number of persons is affected by Mr. Sheldon's decision that it is not likely to go unchallenged, especially as the nurses' claim to a vote has been allowed elsewhere.

---

The work accomplished during the war by the London No. 1 Voluntary Aid Detachment deserves special recognition from the fact that its duties were of a pioneer nature. It was the first to be formed. It had to find out what to do and how to do it. It had to train officers, organise drills, improvise implements and make members efficient who were already fully occupied in other directions and could only spare odds and ends of time or surrender their sleep. The little volume edited by Stanley Unwin, Section Leader, on "The Work of V.A.D. London during the War" (George Allen and Unwin, 5s.), will be read with great interest by all our readers, and they are numerous, who came into connection with this famous detachment. The chapter contributed by Sir James Cantlie, K.B.E., founder and commandant, is particularly illuminating, for it describes the inception of ambulance work and traces the movement back to the year 1878, when the ambulance department of the Order of St. John was being formed under Surgeon Major Shepherd, shortly after killed in the Zulu war.

---

At the Preston Royal Infirmary the scale of salaries has again been raised. Probationers are now to receive £20, £25, £30, and in their fourth year £40. The hospital is undergoing extension and the nursing staff is to be increased. The boon of a day's rest once a week was extended to the nurses last December, and it is highly satisfactory to learn that it is working very well and is much appreciated. In these circumstances Miss Marks should have no difficulty in securing the additional probationers she requires. We note that she is prepared to receive applications from educated girls of twenty and upwards.

---

The presentation of a handsome gift to Miss A. E. Howe, the retiring matron of the Children's Infirmary, Lambeth, was made the occasion for a very pleasant ceremonial of leave-taking in which Mr. and Mrs. Olsen, the retiring master and matron of the Princes Road Workhouse, which has now been closed, took part. Mr. Morris, the superintendent of the Children's Establishment, spoke very warmly of the tender solicitude and devotion with which Miss Howe had ministered to the little ones who came under her care at the Infirmary, and summed up her dealings with her staff with the words "She is a gentlewoman—the highest compliment that can be paid to one of her sex." Allusion was made to the difficulties experienced during air-raids, when her quiet, self-restrained manner among the children gave them confidence and banished panic. We could wish that the cause of her retirement from her long labours of love were not ill-health, but the exceptional strain of the war years has left its mark on many who bore the brunt at home.

---

The considerable number of resignations at Southwark Infirmary have led the Infirmary Visiting Committee to make inquiries to find out what was the reason. The first matter they took in hand was the dietary. At present the nurses, as well as the other officers, are practically on war diet. The allowance of meat per head is only two pounds a week, and should the staff be out for a half-day or a day, when they return at night all there is for them is either a little cold soup or rice pudding. Mrs. Hale, who presides over the committee, stated that the nurses had to spend their own money on food. With a view to relieving matters the guardians have decided to double the meat allowance. Then, again, the allowance of milk is to be increased to a pint a day, and the present system of issuing half the ration in the form condensed is to be abolished, while the sugar ration is raised from six to eight ounces. It is hoped that in course of time other improvements will take place, but in view of the scarcity and dearness of certain commodities, it is impossible to get back at present to the pre-war scale of diet.

---

The increased health powers conferred by the Ministry of Health on local authorities are beginning to signal the demise of many voluntary organisations which hitherto have been doing various branches of work which local authorities left undone. Now the Isle of Dogs Infant Welfare Association, which is one of the pioneer bodies to undertake maternity and child welfare work in London, is scheduled for municipalisation, for the Poplar Borough Council proposes to take the organisation over from Miss Wintour. The work has been done by the Association for some fourteen years, and the Borough Council declares it to have been highly successful. The clinics have been of great value, and the attendance of mothers at the centres is higher now than at any time. The Borough Council Committee, which has interviewed the Committee of the Association, reports that it is impressed by the evident confidence of the mothers and affection for those who have been carrying on the work. The fact that the Council recognises that the work has been well done is proved by the proposal to continue the centres on the same lines.

